# Femtosecond laser-assisted cataract surgery in a spherophakic lens: An unusual case report

**DOI:** 10.1097/MD.0000000000017426

**Published:** 2019-11-01

**Authors:** Danmin Cao, Yong Wang, Rong Lei, Mahmood Khan, Li Wang

**Affiliations:** a Wuhan Aier Eye Hospital, Aier Eye Hospital Group, Wuchang District, Wuhan; b Aier School of Ophthalmology, Central South University, Tianxin District, Changsha, Hunan Province, China; c Cullen Eye Institute, Department of Ophthalmology, Baylor College of Medicine, Houston, TX.

**Keywords:** cataract, femtosecond laser, spherophakic lens, subluxated lens

## Abstract

**Rationale:**

Spherophakia is a rare diagnosis which is often associated with a shallow anterior chamber, angle-closure glaucoma, lens subluxation, and lenticular myopia. When cataracts occur with subluxation of the lens, vision is often markedly affected. This often presents surgeons with a unique challenge of maintaining good visual outcomes while minimizing potential complications.

**Patient concerns:**

A 48-year-old female was referred for ophthalmological assessment due to decreased vision in the left eye. In the left eye, the best-corrected visual acuity at distance was 20/125 with manifest refraction of −6.5D + (−0.75) D × 118°. The slit lamp examination showed iridodonesis and a significant nuclear cataract (C3N3) with tremor in the left eye. After pupil dilation, a subluxated lens, weak zonules, and “fake golden ring” within the lens was noted.

**Diagnosis:**

Due to the patient's symptoms, examination results, she was diagnosed with cataract, subluxation of the lens and spherophakia in left eye.

**Interventions:**

The patient underwent an uneventful femtosecond laser-assisted cataract surgery (Alcon Fort Worth, TX). The laser was able to perform a circular free-floating anterior capsulotomy and easy lens fragmentation.

**Outcomes:**

There were no postoperative complications. At 3 months postoperatively, the uncorrected visual acuity was 20/25, and the manifest refraction was −0.25 D − 0.75 D × 148° with the corrected distance visual acuity of 20/16.

**Lessons:**

Femtosecond laser-assisted cataract surgery is an effective approach for cataract surgery in patients with subluxated and spherophakic lenses, with the benefits of causing minimal further zonular damage and easy lens fragmentation.

## 1 Introduction

Spherophakia is a rare diagnosis which is often associated with a shallow anterior chamber, angle-closure glaucoma, lens subluxation, and lenticular myopia.^[1]^ In this condition, the crystalline lens demonstrates a reduced equatorial diameter and an expanded anterior-posterior diameter.^[2]^ When cataracts occur with subluxation of the lens, vision is often markedly affected. This often presents surgeons with a unique challenge of maintaining good visual outcomes while minimizing potential complications. Femtosecond laser-assisted cataract surgery (FLACS) provides surgeons with a better option in these cases, since this technique is not dependent on zonular countertraction in the creation of a continuous curvilinear capsulorhexis (CCC).^[3]^ In this case, we describe the intraoperative surgical techniques in a patient who presented with a subluxated spherophakic lens and underwent femtosecond laser-assisted cataract surgery.

This case report was approved by the ethics committee of the WuHan Aier Eye Hospital, Hubei, China, and the informed consent form was signed by patient.

## 2 Case report

**Figure 1:**
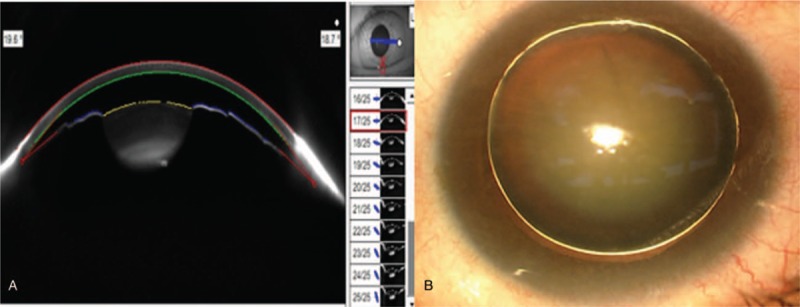
(A) A micro spherophakic lens was confirmed on the ocular anterior segment image (Pentacam), it shows shallow anterior chamber and thick lens. (B) A subluxated lens with zonular dialysis, and a fake golden ring within the lens.

A 48-year-old female was referred for ophthalmological assessment due to decreased vision in the left eye. In the left eye, the best-corrected visual acuity at distance was 20/125 with manifest refraction of −6.5 D + (−0.75) D × 118°, and the intraocular pressure was 19.3 mm Hg, using the optical biometer (Lenstar, Haag-Streit AG, Köniz, Switzerland), the mean corneal curvature, central corneal thickness, axial length, anterior chamber depth, lens thickness, and horizontal corneal diameter was 45.24 D, 553 μm, 24.38 mm, 1.89 mm, 5.96 mm, and 12 mm, respectively. The corneal endothelial cells counts was 2463 cells/mm^2^. The slit-lamp examination showed iridodonesis and a significant nuclear cataract (C3N3) with tremor in the left eye. A spherophakic lens was confirmed on ocular anterior segment imaging (Pentacam, Oculus, Optikgeräte GmbH, Wetzlar, Germany) (Fig. 1A), it shows shallow anterior chamber and thick lens. After pupil dilation, a subluxated lens, weak zonules, and “fake golden ring” within the lens was noted (Fig. 1B). The ratio of optical nerve cup/disk was 0.3. No history of traumar pain was reported and examination revealed no signs of retinal detachment or uveitis.

**Figure 2:**
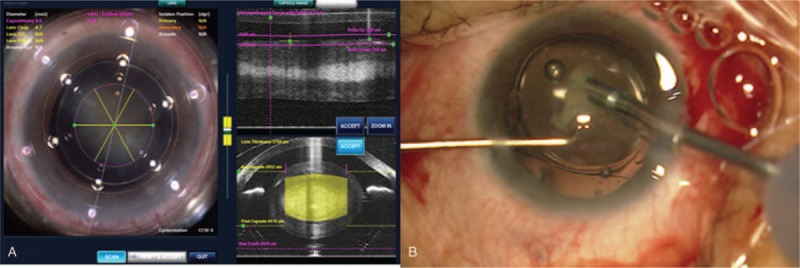
(A) The OCT image of the eye with outlined boundaries of the cornea and lens capsule. The capsulotomy pattern and lens fragmentation pattern are guided by the LenSx laser system (Alcon Laboratories Inc). (B) The IOL was implanted in the bag before the residual fragment were removed, as described in the “IOL-shell technique.” IOL = intraocular lens, OCT = optical coherence tomography.

Lens extraction and intraocular lens (IOL) implantation were subsequently performed using the LenSx laser system (Alcon Laboratories Inc, 2.3, Fort Worth, TX). After patient interface docking, a 5.0 mm diameter capsulotomy with 6 μJ laser energy and 5 μm spot separation was performed and the anterior segment optical coherence tomography (OCT) showed dislocation of the spherophakic lens (Fig. 2A). A cylinder and chop pattern was used for lens fragmentation with 12 μJ laser energy and 10 μm spot separation. After the completion of the laser procedure, a 2.2 mm corneal incision was created with a scalpel and. phacoemulsification was completed using the Infiniti Vision System (Alcon Inc, Fort Worth, TX). During phacoemulsification, the capsulotomy was found to be complete and free-floating. Four disposable nylon capsular hooks were placed on the capsulorhexis edge to support the lens and stabilize the capsular bag during phacoemulsification.^[4]^ Gentle hydrodissection and hydrodelineation were performed carefully to evacuate cavitation bubbles. The nucleus was then separated along the femtosecond cleavage planes and aspirated with the phacoemulsification probe. After most of the fragments were removed, a capsular tension ring was implanted to stretch the posterior capsule and further stabilize the capsular bag,^[5]^ and then a foldable Akreos Adapt AO *IOL* (Bausch and Lomb Inc, Rochester, New York) was implanted. To reduce the risk for posterior capsular rupture, the IOL was implanted in the bag before the residual fragment was removed, as described in the “IOL-shell technique” (Fig. 2B).^[6]^

**Figure 3:**
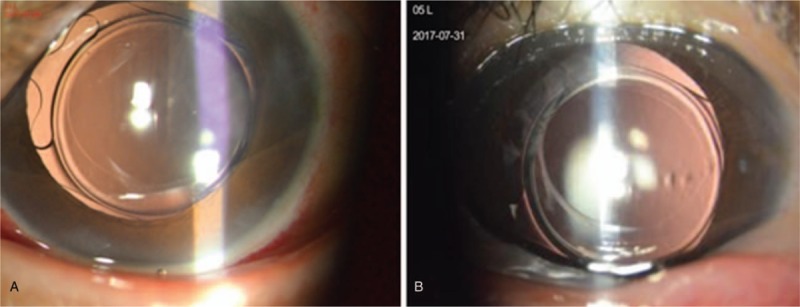
(A) Well-centered IOL with a 360° capsule overlap at 1 d. (B) 3 mo postoperatively. IOL = intraocular lens.

On postoperative day 1, the uncorrected visual acuity (UCVA) was 20/30, and the IOL was well-centered with a 360° capsule overlap (Fig. 3A). At 3 months postoperatively, the UCVA was 20/25, and the manifest refraction was −0.25 D − 0.75 D × 148° with corrected distance visual acuity of 20/16. The IOL was noted to be well centered (Fig. 3B). Post-op 3 months, the corneal endothelial cells counts were 2242 cells/mm^2^, and the central anterior chamber depth was 3.84 mm.

## 3 Discussion

Spherophakia is an uncommon condition usually associated with syndromes such as Weill-Marchesani, Marfan, or Alport syndrome. Spherophakic cataracts with a shallow anterior chamber and subluxated lens present a high risk of causing intraoperative complications.^[7]^ Performing a CCC and lens fragmentation are 2 critical steps for cataract surgery. However, a subluxated lens with zonular loss often makes it difficult to perform a manual capsulorhexis and fragmentation. In our case, we used FLACS to create a circular capsulotomy centered on the subluxated capsular bag in a closed chamber and lens fragmentation with no additional zonular stress. The femtosecond laser significantly reduces potential complications by reducing the zonular stress that would otherwise be created with a manual capsulorhexis.^[8]^ Crema et al reported that the femtosecond laser does not depend on zonular support to create a capsulotomy in patients with Marfan syndrome with mild, moderate, and severe lens subluxation.^[9]^

One of the advantages of the laser software is the option of manually repositioning the capsulotomy so that it can be better centered on the subluxated lens. The software also allows change in the capsulotomy and lens fragmentation size which is useful for the spherophakic lens, as the lens is thicker and more difficult to fragment than normal crystalline lens.^[10]^ Femtosecond laser can accurately set the depth of nucleus fragmentation under the guidance of OCT imaging, which helps in performing effective nuclear separation and phacoemulsification. In this case, we also used the IOL-shell technique, as the IOL can act as a suitable mechanical barrier to keep the posterior capsular lens in a stable and safe position and prevent the adverse impact of ultrasound waves or unstable fluidity on the posterior lens capsule.^[6]^ In this case, the Akreos Adapt AO IOL was used because it is an aspheric IOL that has been specifically designed with zero spherical aberration in the event capsule contraction and IOL tilt occur.

To the best of our knowledge, this is the only report of FLACS combined with a subluxated and spherophakic cataract. Our findings demonstrated that femtosecond laser is superior in terms of ease and speed for creation of the capsulorrhexis in these eyes. The IOL-shell technique may help make this surgery safer in this unique patient population, as evidenced in this case. The safety and limits of the FLACS in patients with subluxated spherophakic cataracts will need to be evaluated in additional studies.

## Acknowledgment

The authors thank Xiao Wang for collecting the patient's follow up data.

## Author contributions

**Conceptualization:** Danmin Cao, Yong Wang, Rong Lei, Li Wang.

**Data curation:** Danmin Cao, Rong Lei, Mahmood Khan.

**Formal analysis:** Danmin Cao, Yong Wang, Rong Lei.

**Funding acquisition:** Danmin Cao, Yong Wang, Li Wang.

**Investigation:** Danmin Cao, Yong Wang, Li Wang.

**Methodology:** Danmin Cao, Yong Wang, Li Wang.

**Project administration:** Danmin Cao, Yong Wang, Rong Lei, Li Wang.

**Resources:** Danmin Cao, Yong Wang, Mahmood Khan.

**Software:** Yong Wang, Rong Lei, Mahmood Khan, Li Wang.

**Supervision:** Danmin Cao, Yong Wang, Mahmood Khan.

**Validation:** Danmin Cao, Yong Wang, Rong Lei, Li Wang.

**Visualization:** Danmin Cao, Yong Wang.

**Writing – original draft:** Danmin Cao.

**Writing – review and editing:** Danmin Cao, Yong Wang, Rong Lei, Mahmood Khan, Li Wang.
